# Membrane Phospholipids and Polyphosphates as Cofactors and Binding Molecules of SERPINA12 (vaspin)

**DOI:** 10.3390/molecules25081992

**Published:** 2020-04-24

**Authors:** Catherine A. Tindall, Sebastian Dommel, Veronika Riedl, David Ulbricht, Stefanie Hanke, Norbert Sträter, John T. Heiker

**Affiliations:** 1 Institute of Biochemistry, Faculty of Life Sciences, Leipzig University, 04103 Leipzig, Germany; catherine.tindall@uni-leipzig.de (C.A.T.); sebastian.dommel@medizin.uni-leipzig.de (S.D.); veronika.riedl@uni-leipzig.de (V.R.); david.ulbricht@medizin.uni-leipzig.de (D.U.); 2 Institute of Bioanalytical Chemistry, Center for Biotechnology and Biomedicine, Leipzig University, 04103 Leipzig, Germany; stefanie.hanke@uni-leipzig.de (S.H.); strater@bbz.uni-leipzig.de (N.S.); 3 Helmholtz Institute for Metabolic, Obesity and Vascular Research (HI-MAG) of the Helmholtz Zentrum München at the University of Leipzig and University Hospital Leipzig, 04103 Leipzig, Germany

**Keywords:** serpin, protease, cofactor, polyphosphates, phosphatidylinositol phosphates, membrane lipids

## Abstract

Visceral adipose tissue derived serine protease inhibitor (vaspin) is a member of the serpin family and has been shown to have beneficial effects on glucose tolerance, insulin stability as well as adipose tissue inflammation, parameters seriously affected by obesity. Some of these effects require inhibition of target proteases such as kallikrein 7(KLK7) and many studies have demonstrated vaspin-mediated activation of intracellular signaling cascades in various cells and tissues. So far, little is known about the exact mechanism how vaspin may trigger these intracellular signaling events. In this study, we investigated and characterized the interaction of vaspin with membrane lipids and polyphosphates as well as their potential regulatory effects on serpin activity using recombinant vaspin and KLK7 proteins and functional protein variants thereof. Here, we show for the first time that vaspin binds to phospholipids and polyphosphates with varying effects on KLK7 inhibition. Vaspin binds strongly to monophosphorylated phosphatidylinositol phosphates (PtdInsP) with no effect on vaspin activation. Microscale thermophoresis (MST) measurements revealed high-affinity binding to polyphosphate 45 (K_D_: 466 ± 75 nM) and activation of vaspin in a heparin-like manner. Furthermore, we identified additional residues in the heparin binding site in β-sheet A by mutating five basic residues resulting in complete loss of high-affinity heparin binding. Finally, using lipid overlay assays, we show that these residues are additionally involved in PtdInsP binding. Phospholipids play a major role in membrane trafficking and signaling whereas polyphosphates are procoagulant and proinflammatory agents. The identification of phospholipids and polyphosphates as binding partners of vaspin will contribute to the understanding of vaspins involvement in membrane trafficking, signaling and beneficial effects associated with obesity.

## 1. Introduction

The visceral adipose tissue derived serine protease inhibitor (vaspin, SERPINA12 according to the serpin nomenclature [1]) was first identified in the Otsuka Long-Evans Tokushima fatty (OLETF) rat type 2 diabetes model [2]. Vaspin serum levels are significantly higher in patients with obesity and type 2 diabetes [3,4] and multiple lines of evidence indicate a compensatory and counteracting role in obesity-related disorders and diseases (reviewed in [5]). Vaspin administration improves glucose tolerance and adipose tissue inflammation in obese mice [6,7]. In the brain, central application of vaspin reduced food intake and improved insulin sensitivity by regulating hepatic glucose production and insulin signal transduction [8,9,10]. On the cellular level, vaspin was shown to exhibit anti-apoptotic and anti-atherogenic potential in endothelial and smooth muscle cells [11,12,13] as well as anti-inflammatory effects in adipocytes [14,15] and skin [16]. In vivo, transgenic overexpression of vaspin protects mice from diet-induced adipose tissue inflammation while knockout mice exhibit deterioration of metabolic functions in obesity [17]. Further anti-inflammatory effects in the liver have been in part associated with vaspins interaction with the ER chaperon GRP78 [17].

There are two protease targets for vaspin known so far, both kallikrein-related peptidases, kallikrein 7 (KLK7) and kallikrein 14 (KLK14) [7,18]. Vaspin inhibits target proteases by the classical serpin suicide-substrate mechanism. The reactive center loop (RCL) is exposed on top of the vaspin molecule and serves as bait for the target proteases. After RCL cleavage, the RCL is inserted into the central β-sheet A and the protease is translocated to the bottom of the vaspin molecule forming a covalent complex. The active center of the protease is distorted and hence the protease becomes inactivated [19]. Both, KLK7 and KLK14 cleave the vaspin RCL after Met^378^ [18,20]. Improvement of glucose tolerance is dependent on vaspins serpin function and inhibition of KLK7 by vaspin is hypothesized to prolong insulin action in the circulation and thereby contributes to improve glucose tolerance in vivo, as insulin was found to be a substrate of KLK7 [7]. Furthermore, knockout of KLK7 in adipose tissue preserved insulin sensitivity in obesity by counteracting adipose tissue inflammation under high-fat diet in vivo [21]. Both kallikrein proteases are also involved in the process of skin desquamation. Dysregulated proteolytic activity, especially of KLK7, is a major cause for inflammatory skin diseases such as Netherton syndrome [22] and psoriasis [23]. Additionally, vaspin is highly expressed in human skin and may contribute to regulation of kallikrein activities in this tissue [24].

Previously, we demonstrated binding of vaspin to the negatively charged glycosaminoglycan heparin. Binding of the glycosaminoglycan chain accelerates vaspin/KLK7 complex formation via a bridging mechanism [25], bringing serpin and protease into close proximity and preferential orientation [26]. In contrast to other serpins such as heparin cofactor II (SERPIND1) [27], plasminogen activator inhibitor 1 (PAI, SERPINE1) [28] or antithrombin (AT3, SERPINC1) [29], which bind heparin at the opposite side of the serpin molecule (in helix D), heparin binds to vaspin at a unique positively charged patch located in the central β-sheet A. Residues Arg^211^ and Lys^359^ are key residues mediating high-affinity heparin binding by vaspin (K_D_: 21.6 ± 2.5 nM) [25]. Furthermore, we have provided evidence that a substantial amount of secreted vaspin is bound to heparan sulfates of the extracellular matrix [25].

In this study, we investigated the interaction of vaspin with two other components of the cell surface, phosphatidylinositol phosphates (PtdInsP) and polyphosphates (polyP). PtdInsPs are important components of the plasma membrane and a group of negatively charged phospholipids with different phosphorylation patterns of the inositol head group [30]. They are involved in diverse functions such as membrane reorganization, endocytosis and signal transduction [31]. PolyPs on the other hand are highly negatively charged linear polymers of up to 800 monophosphate units in mammalian cells [32]. These have been shown to bind to serpins and accelerate protease inhibition in a heparin-like manner, e.g., for protease C1s by serpin C1 esterase inhibitor [33]. Furthermore, polyPs were shown to mediate procoagulant as well as proinflammatory effects [34]. The identification of new vaspin-binding molecules associated with membrane trafficking, and cellular signaling may help better understanding molecular mechanisms of cellular and tissue-specific effects reported for vaspin.

## 2. Results

### 2.1. Vaspin Binds Membrane Phospholipids Phosphatidylinositol Phosphates and Phosphoserine

**Figure 1:**
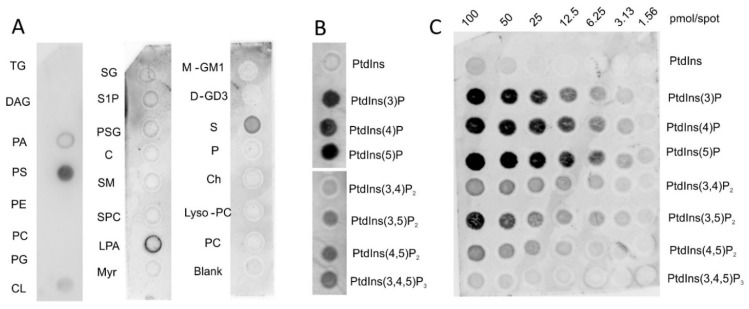
Vaspin affinity for immobilized membrane lipids. Shown are lipid overlay assays analyzing binding of vaspin to a variety of membrane lipids with 100 pmol/spot of lipid immobilized on each spot and 1 µg/mL vaspin was used for incubation of (**A**) membrane lipid and sphingo lipid strips, (**B**) PtdIns lipid strip and (**C**) PtdIns array with serial dilutions of different PtdInsPs from 100 pmol down to 1.56 pmol/spot as indicated. TG: triglyceride, DAG: diacylglycerol, PA: phosphatidic acid, PS: phosphatidylserine, PE: phosphatidylethanolamine, PC: phosphatidylcholine, PG: phosphatidylglycerol, CL: cardiolipin, SG: sphingosine, S1P: sphingosine-1-phosphate, PSG: phytosphingosine, C: ceramide, SM: sphingomyelin, SPC: sphingosylphosphorylcholine, LPA: lysophosphatidic acid, Myr: myriosine, M-GM1: monosialoganglioside-GM1, D-GD3: disiaganglioside-GD3, S: sulfatide, P: psychosine, Ch: cholesterol, PtdIns: phosphatidylinositol, PtdIns(3)P: phosphatidylinositol (3)-phosphate, PtdIns(4)P: phosphatidylinositol (4)-phosphate, PtdIns(5)P: phosphatidylinositol (5)-phosphate, PtdIns(3,4)P_2_: phosphatidylinositol (3,4)-bisphosphate, PtdIns(3,5)P_2_: phosphatidylinositol (3,5)-bisphosphate, PtdIns(4,5)P_2_: phosphatidylinositol (2,4)-bisphosphate, PtdIns(3,4,5)P_3_: phosphatidylinositol (3,4,5)-trisphosphate.

Vaspin is a highly basic protein with an isoelectric point (pI) of 9.3 and we have previously reported its mechanism of binding to heparin and heparan sulfate proteoglycans of the extracellular matrix [25]. Here, we investigated interactions of vaspin with negatively charged components of the plasma membrane, focusing on phospholipids and other lipid species of the cell membrane. Simple lipid overlay assays demonstrated specific binding of vaspin to phosphatidylserine (PS; Figure 1A) and phosphoinositides, especially monophosphorylated phosphatidylinositol phosphates (PtdInsPs) (Figure 1B). Weak signals were obtained for cardiolipin (CL) and sulfatide (S), while diacylglycerol (DAG), phosphatidyl-ethanolamine (PE), -cholin (PC), -glycerol (PG), as well as cholesterol (Ch) and many tested sphingolipids gave no detectable signal (Figure 1A).

Using a lipid array strip with increasing amounts of immobilized PtdInsPs confirmed these results showing saturable binding especially for the three monophosphorylated PtdInsPs within the concentration range tested (Figure 1C). Medium affinity was detected for PtdIns(3,5)P_2_ and weak binding for the other two bisphosphorylated PtdInsPs PtdIns(3,4)P_2_ and PtdIns(4,5)P_2_. PtdIns(3,4,5)P_3_ was bound with minimal affinity. There was no detectable signal for phosphatidylinositol indicating importance of the number and position of phosphate moieties for vaspin binding.

### 2.2. PtdInsPs Do Not Affect KLK7 Inhibition by Vaspin

**Figure 2:**
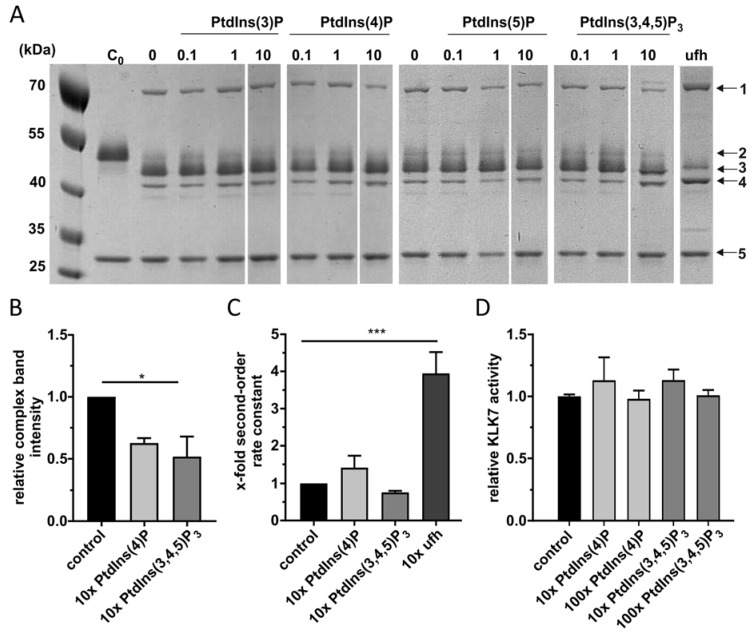
Influence of PtdInsPs on vaspin/KLK7 complex formation and KLK7 activity. (**A**) SDS-PAGE analysis of vaspin/KLK7 complex formation. Vaspin wt was incubated with x-fold excess of PtdInsPs (0.1, 1 and 10-fold) or unfractionated heparin (ufh, 10-fold) as indicated. Notable and indicated bands are: 1-vaspin-protease complex; 2-full-length vaspin; 3-*N*-terminally cleaved vaspin; 4-RCL- and *N*-terminally cleaved vaspin; 5-KLK7. KLK7 was incubated with vaspin (at a molar ratio 3:1) for 2 min. C_0_: control reaction after t = 0 min. (**B**) Densitometric quantification of complex formation with and without PtdInsPs or ufh. Presented is the relative increase of complex band intensity as x-fold over control (vaspin without PtdInsP). (**C**) Inhibition of KLK7 by vaspin was measured under pseudo-first-order conditions (ligand/serpin ratio of 10). Presented is the relative increase in second-order rate constant as x-fold over control (without PtdInsP or heparin). (**D**) KLK7 activity was measured in presence of 10 or 100-fold excess of PtdInsPs. Presented is the relative KLK7 activity as x-fold over control (without PtdInsPs). Data are presented as means ± SEM. Statistical significance was determined by one-way ANOVA followed by Dunnett’s post-hoc test. * *p* < 0.05, *** *p* < 0.001.

Since the most efficient binding was observed for monophosphorylated PtdInsPs, we studied potential regulatory effects of the latter on the inhibition reaction of vaspin and target protease KLK7 by analyzing complex formation via SDS-PAGE. As previously shown, vaspin forms a stable complex with KLK7 with a molecular weight of 70 kDa. Furthermore, *N*-terminally and reactive center loop (RCL) cleaved vaspin bands appear at molecular weights of 44 and 42 kDa (Figure 2A). In contrast to heparin serving as a positive control, we did not observe any accelerating effect on complex formation for all PtdInsPs tested. Densitometric quantification for 10-fold PtdInsP rather revealed a decrease in complex formation (Figure 2B).

Interestingly, we observed more RCL-cleaved vaspin in the presence of PtdIns(3,4,5)P_3_ although only weak binding indicated by the lipid overlay assay. Therefore PtdIns(3,4,5)P_3_ seems to affect RCL cleavage by binding to KLK7. Additionally, the estimated stoichiometry of inhibition (SI) was increased 3-fold. In order to more precisely evaluate PtdInsPs effects on vaspins inhibitory activity, we measured KLK7 inhibition rates in the presence of 10-fold excess of PtdInsPs. Heparin again served as an accelerating positive control. These data confirmed the gel-based results, as PtdInsPs did not increase the second-order rate constant of KLK7 inhibition by vaspin (Figure 2C), while heparin significantly increased the second-order rate constant 5-fold as shown before [25]. 

To exclude regulatory effects of PtdInsPs on KLK7 we measured KLK7 activity in the presence of different concentrations of PtdInsPs. We did not observe any effect of PtdInsPs on KLK7 activity (Figure 2D).

### 2.3. High-Affinity PolyP_45_ Binding Accelerates Vaspin-KLK7 Complex Formation 

**Figure 3:**
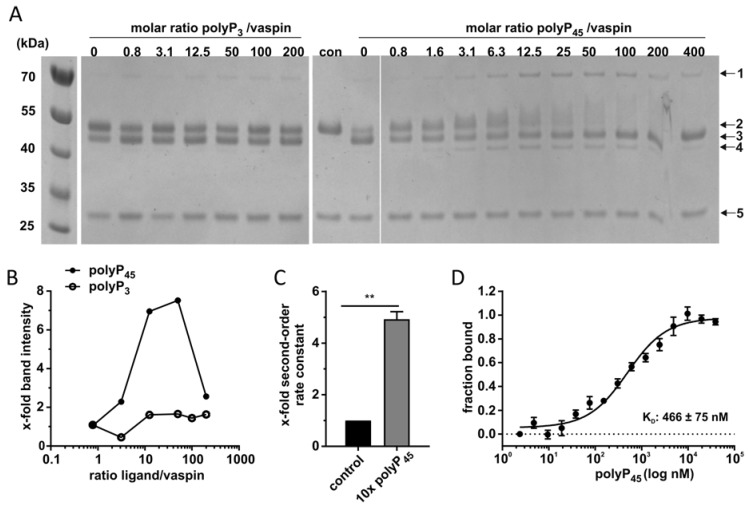
Influence of polyphosphates on complex formation. (**A**) Shown is complex formation of vaspin with KLK7 (protease/serpin molar ratio 3:1) with increasing concentrations of polyP_3_ and polyP_45_ (0.8−400-fold as indicated) after 1 min. Notable and indicated bands are: 1-vaspin-protease complex; 2-full-length vaspin; 3-*N*-terminally cleaved vaspin; 4-RCL- and *N*-terminally cleaved vaspin; 5-KLK7. (**B**) Densitometric quantification of complex band intensities in relation to ligand/vaspin ratio of SDS gels. (**C**) Inhibition of KLK7 by vaspin under pseudo first-order conditions in presence of polyP_45_ (polyP/serpin ratio of 10:1). Presented is the second-order rate constant as x–fold over control (without polyP). (**D**) Binding of polyP_45_ to fluorescently labeled vaspin in submicromolar range. The curve was derived from the measurement of the thermophoretic mobility after titration of polyP_45_ to a constant vaspin concentration. Data are presented as means ± SEM. Statistical significance was determined by Student’s two-tailed *t*-test. ** *p* < 0.01.

Previous studies have demonstrated polyphosphates activate the inhibitory action of serpin towards C1s with submicromolar affinity (K_D_: 450 nM) in a heparin-like manner [33]. Here, we analyzed vaspin binding to polyphosphates with different length (polyP_3_ and polyP_45_) and the potential acceleration of the inhibition reaction for KLK7. The triphosphate did not affect complex formation while a clear dose-dependent increase in complex band intensity was detected up to an excess of 100-fold of polyP_45_ (100 µM, Figure 3A/B). With higher amounts of polyP_45_, the complex band intensity decreased again, revealing a bell-shaped dose-response curve, as previously observed for heparin. Furthermore, a clear shift in electrophoretic mobility was observed for vaspin in the presence of increasing polyP_45_ concentrations. In line with these observations, the second-order rate constant for KLK7 inhibition increased 5-fold in the presence of polyP_45_ (Figure 3C). These findings demonstrate that longer polyphosphate chains are able to accelerate protease inhibition by vaspin via the bridging mechanism.

To determine the affinity of vaspin for polyP_45_ we performed microscale thermophoresis. This revealed high affinity binding with a dissociation constant (K_D_) of 466 ± 75 nM for the interaction of vaspin with polyP_45_ (Figure 3D).

### 2.4. PtdInsPs and Heparin Share the Same Binding Site

**Figure 4:**
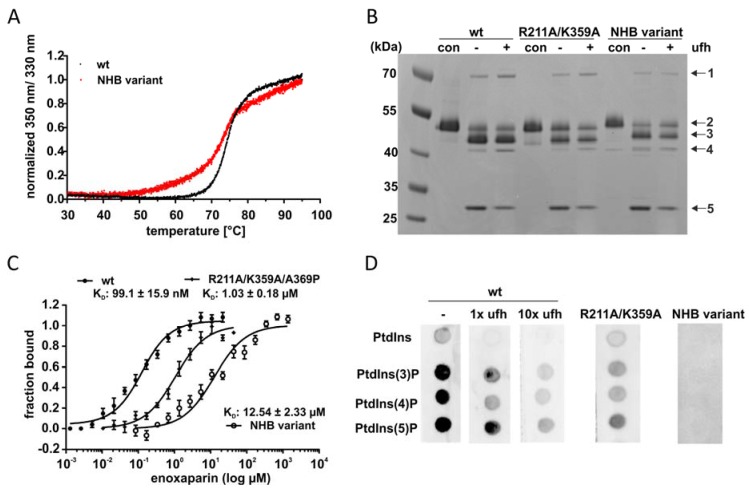
Investigation of the PtdInsP binding site using a non-heparin binding (NHB) vaspin variant. (**A**) Thermal stability of wt vaspin and NHB variant (K188A/K131A/R211A/K359A/R363A). Denaturation was observed by nanoDSF plotting the intrinsic tryptophan and tyrosine fluorescence ratio of 350 nm/330 nm against temperature. (**B**) Complex formation of wt vaspin, R211A/K359A and NHB variant in the absence (−) and presence of heparin (+; heparin/vaspin ratio of 10:1) for 1 min. Notable and indicated bands are: 1-vaspin-protease complex; 2-full-length vaspin; 3-*N*-terminally cleaved vaspin; 4-RCL- and *N*-terminally cleaved vaspin; 5-KLK7. (**C**) Binding of the low-molecular weight heparin clexane to wt vaspin, R211A/K359A and NHB variant. Data from the wt and R211A/K359A was originally published in the Journal of Biological Chemistry: Ulbricht D, Oertwig K, Arnsburg K, Saalbach A, Pippel J, Strater N and Heiker JT. Basic Residues of β-Sheet A Contribute to Heparin Binding and Activation of Vaspin (Serpin A12). *J Biol Chem*. 2017, 292, 994–1004, © the American Society for Biochemistry and Molecular Biology. Curves were derived from changes in fluorescence (wt or R211A/K359A) or thermophoretic mobility (NHB) after titration of enoxaparin to a constant vaspin concentration. (**D**) Lipid strips incubated with wt vaspin (alone (−) or in presence of heparin, with a molar ratio serpin/heparin of 1:1 or 10:1 as indicated) and vaspin variants. NHB: non-heparin binding variant, con: control, ufh: unfractionated heparin.

Previously, we identified key residues mediating high-affinity heparin binding in Arg^211^ and Lys^359^ with the R211A/K359A variant exhibiting a 10-fold decrease in heparin affinity. Still, residual heparin binding was still observable indicating that more residues are involved in heparin binding [25]. To investigate whether this basic patch at the central sheet A is also relevant for the interaction with the here newly identified binding molecules, we mutated all basic residues within the heparin binding site. This yielded the K188A/K131A/R211A/K359A/R363A variant (referred to as non-heparin binding (NHB) variant). We first determined thermal stability to exclude altered structural integrity and stability due to the loss of five charged residues. The NHB variant had a less cooperative and sharp melting point compared to the wild type, but the melting temperature was identical (74 °C, Figure 4A) indicating a very stable and folded enzyme structure.

In the following, we analyzed the effect of heparin on complex formation for variants R211A/K359A and NHB. Activity of the two variants without heparin was comparable to wild type (Figure 4B). We observed diminished complex acceleration by heparin for the NHB variant compared to R211A/K359A variant and the wild type (Figure 4B, lane ‘+’).

Additionally, we investigated heparin binding of vaspin and variants by microscale thermophoresis. The dissociation constant of the NHB variant for the low-molecular weight heparin enoxaparin was significantly decreased by 10-fold compared to the R211A/K359A variant and by 100-fold compared to the wild type (12 µM vs. 1 µM vs. 100 nM, respectively; Figure 4C), demonstrating complete loss of high affinity binding to heparin.

To investigate PtdInsP binding, vaspin wild type (free and in presence of heparin) and non-heparin-binding variants were analyzed using lipid overlay assays. The presence of heparin (molar ratios of heparin/vaspin of 1 and 10) and mutations of heparin binding residues (R211A/K359A or NHB variant) decreased vaspin binding to PtdInsPs (Figure 4D). Notably, the presence of an equimolar concentration of heparin (21 nM and thus corresponding to the K_D_ for heparin) already significantly prevented phospholipid binding.

**Figure 5:**
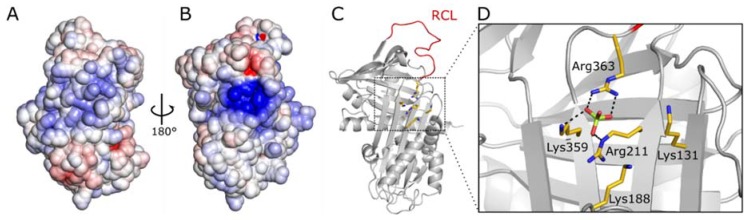
Heparin and phosphate ion binding site of vaspin. (**A**,**B**) Electrostatic potential at the molecular surface of vaspin (PDB 4IF8) [7] as viewed from the opposite sides. A large area of strong positive potential is visible in the orientation depicted in (**B**). Potential values < −8 kT/e are colored in red and values > +8 kT/e in blue. The electrostatic potential was generated with the program APBS [35]. The protein orientation in (**B**) is the same as that of (**C**), showing the protein fold and the basic residues generating the strong positive electrostatic potential. The reactive center loop (RCL, red) is flexible in the crystal structure and modeled here for orientation. Five basic resides are present in the area of the distinct positive potential (**C**) and a sulfate ion is coordinated by three side chains (**D**).

The five basic residues, which were mutated in the NHB variant, generate a distinct strong positive electrostatic potential (Figure 5). In the crystal structure of vaspin, a sulfate ion is bound to this site, coordinated by Lys^188^, Arg^211^ and Arg^363^ (Figure 5D) [7]. Vaspin was crystallized in the presence of 0.1 M ammonium sulfate. It appears likely that one of the phosphate ions of polyphosphates or the PtdInsPs binds in a similar manner to this binding site.

## 3. Discussion

Previous work has already provided evidence for vaspin binding to heparin and heparan sulfate proteoglycans in the extracellular matrix [25]. Here, we investigated phosphorylated membrane lipids as potential novel binding partners for vaspin. Our study revealed strong binding to monophosphorylated PtdInsPs, while bis- as well as triphosphorylated PtdInsPs were weakly bound and binding of unphosphorylated PtdIns was not observable. This indicates that binding of vaspin to PtdInsPs is both dependent on the presence and localization of the charged phosphate moiety of the phospholipid, as multiple phosphate groups, e.g., of PtdIns(3,4,5)P_3_ rather decreased affinity. We tried to determine affinity by MST measurements using PtdInsPs up to concentrations of 50 µM, but could not obtain binding curves (data not shown). These observations together with heparin competition in the lipid overlay assay indicate affinity for PtdInsPs in the higher µM range. These findings are in line with previously reported affinity of protein C inhibitor (PCI, SERPINA5) for PtdInsP [36]. So far, vaspin and PCI are the only serpins, which were shown to bind to membrane lipids and both with highest affinity for monophosphorylated PtdInsPs. Other heparin binding serpins such as α1-antitrypsin (A1AT, SERPINA1) and antithrombin (AT3, SERPINC1) do not bind to membrane lipids [36]. Phospholipid binding is often mediated through specific domains such as pleckstrin homology or phox homology domains, which preferably bind bis- and triphosphorylated PtdInsPs (reviewed in [37]). Although vaspin does not possess such domains, we provide experimental evidence that monophosphorylated phospholipid binding is mediated via basic residues of the heparin binding site of vaspin. Both, the presence of heparin and mutations of heparin-binding basic residues decreased affinity to PtdInsPs. Wahlmüller et al. as well as Malleier et al. also demonstrated the involvement of the heparin binding site of PCI in membrane lipid binding [36,38]. Thus, our results provide strong evidence that the heparin binding site of vaspin is involved in PtdInsP binding with electrostatic interactions and selective stereospecific recognition of the PtdInsP headgroup mediating vaspin binding. PtdInsPs recognition without a specific domain has been previously shown for a variety of other proteins, e.g., the MARCKS (myristoylated alanine-rich C-kinase substrate) proteins [39,40], c-Src (cellular sarcoma non-receptor protein tyrosine kinase; [41]) or GAP43 (growth-associated protein 43; [40,42]).

The biological function of PtdInsPs is diverse and ranges from signal transduction to membrane transport (reviewed in [31]) with phospholipids recruiting proteins to the plasma membrane. Dysregulation of PtdInsP dynamics or impairment of protein binding to PtdInsPs contribute to human pathologies such as cancer or diabetes [43,44]. The interaction of proteins with membrane lipids induces recruitment and activation of the endocytic and secretory system [45,46,47] where PtdInsPs are distinctly enriched in specific cellular compartments (reviewed in [37]). Intracellular trafficking is mediated by PtdIns(4)P, representing the regulatory phospholipid in the Golgi apparatus, while PtdIns(3)P is mainly located in the endosomes [48]. Both serve as co-receptors for the recruitment of AP-1 and AP-2 clathrin adaptors [49] and therefore are relevant during transfer from the Golgi to the plasma membrane and subsequent secretion to the extracellular space. PtdIns(4)P also serves as precursor for PtdIns(4,5)P_2_ [50] and the latter is enriched in the plasma membrane and essential for clathrin-mediated endocytosis. Together with PtdIns(3,4,5)P_3_ it is also involved in transduction of signaling events and activation of intracellular kinases such as protein kinase B (AKT) and phosphoinositol-dependent kinase 1 (PDK1) [51]. Therefore, vaspins interaction with cell surface PtdInsPs may contribute to activation of intracellular signaling events such as the AKT pathway in endothelial [11] and liver cells [17] or adipocytes [52], whether dependent or independent of insulin. This has also been proposed for the PtdInsP-binding serpin PCI, which has been shown to activate the AKT-pathway, acting as an extracellular but also intracellular serpin [36]. It is unclear, whether vaspin effects on intracellular signaling pathways are furthermore mediated by cell-surface receptors. Additionally, intracellular functions of vaspin, as proposed for PCI [36] or as reported for nuclear α1-antichymotrypsin (ACT, SERPINA3) on cell-cycle progression in hepatic cells [53] have not been investigated so far and may also rely on binding of cytoplasmic membrane lipids. Vaspin binding to bis- and triphosphorylated PtdInsPs was rather weak, yet effects on KLK7-mediated RCL cleavage by PtdIns(3,4,5)P_3_ indicate that binding of PtdIns(3,4,5)P_3_ by vaspin may be underestimated. Still, we found no indications that PtdInsPs play a role in accelerating KLK7 inhibition by vaspin. In contrast to other serpins such as antithrombin or heparin cofactor II, no allosteric activation by low-molecular weight cofactors has been described for vaspin so far. This is in line with previous results demonstrating a prerequisite of >20 units of the polysaccharide chain of glycosaminoglycans for the bridging effect and acceleration of the inhibition reaction [25]. This was also described for other serpin-protease combinations such as antithrombin-factor Xa, antithrombin-thrombin and protein Z-dependent protease inhibitor-factor Xa [54,55,56] as a heparin chain length of >20 is necessary for the bridging mechanism. It remains unclear whether interaction of vaspin with KLK7 and PtdInsPs occurs simultaneously in vivo. Together with the established inhibition of target proteases and binding of cell surface receptors, the interaction with membrane lipids has to be considered in future work investigating cellular functions of vaspin.

Polyphosphates are another class of highly and negatively charged molecules. Previous studies have shown that the serpin C1-esterase inhibitor (C1-INH, SERPING1) is activated by polyP_130_ in a dose-dependent manner in contrast to monophosphates [33] and also inhibition of factor VII-activating protease (FSAP) by plasminogen activator inhibitor 1 (PAI, SERPINE1) is increased by polyP_65_ [57]. Here, we reported for the first time high-affinity binding of vaspin to polyP_45_ with a K_D_ of 466 nM, which is comparable to results obtained for C1-INH [33]. KLK7 inhibition was unaffected by short chain polyP_3_ while a dose-dependent increase of complex formation was detected in presence of polyP_45_. The polyP-mediated increase in inhibition rate was equal to the heparin-induced rate-acceleration, as also previously observed for C1-INH [33].

Polyphosphates have been found present in various cell types, such as platelets [58], mast cells [59] and also tumor cells [60]. When platelets become activated, part of the polyP pool is released as short chain polyphosphate polymers of 60–100 residues [34], while others are presented as polyP nanoparticles on the cell surface of activated platelets and mast cells [61]. Acting as protein-like chaperons, they play a protective role in stress-induced protein aggregation and proteostasis (reviewed in [62]). Contrary, they function as procoagulant as well as proinflammatory mediators in activated platelets [34,61] and induce apoptosis in endothelial cells [63]. Expression of vaspin as well as knockout of KLK7 in adipose tissue has been shown to counteract local and systemic inflammation in obesity [17,21]. Here, binding of polyP may guide and accelerate vaspins interaction with target proteases such as KLK7 and thus regulate proinflammatory proteolytic activity. Furthermore, it may also antagonize direct inflammatory action of polyP by acting as a scavenger and preventing interaction with cell surface receptors such as receptor for advanced glycation end products and P2Y1 purinergic receptor [64]. Both consequences of polyP binding may contribute to previously report anti-inflammatory effects in obesity, and experiments using non-binding vaspin variants in future studies will address these potential contributions of interaction with cell surface molecules. So far, a physiological link between polyP, vaspin and potentially obesity has not been described. PolyP concentrations of 850 pmol/mg protein have been measured in human plasma [65]. A recent study, using a new method of determining plasma polyP in cryoprecipitate, for the first time reported plasma polyP levels in a cohort of 200 metabolically healthy subjects in three body mass index categories (normal weight, overweight and obese) [66]. Surprisingly, they found a negative correlation of plasma polyP levels and BMI, yet it remains to be seen, whether this negative correlation holds true in patients suffering from obesity-associated metabolic diseases such as dyslipidemia, hyperglycemia, insulin resistance or diabetes.

Together, we identified novel non-protease binding partners of vaspin. These findings add to the expanding signaling repertoire of this intriguing member of the serpin family and binding to these components should be considered in addition to protease inhibition and cell-surface receptor binding when investigating and interpreting intercellular effects mediated by vaspin in various cells and tissues.

## 4. Materials and Methods

### 4.1. Materials

Human KLK7 and bacterial thermolysin were purchased from Biolegend and R&D systems, respectively. Fluorogenic peptide NFF3 (Mca-RPKPVE-Nva-WR-K(Dnp)-NH_2_) was from AnaSpec and peptide substrate ortho-aminobenzoic acid (Abz)-KLFSSK-glutaminyl *N*-[2,4-dinitrophenyl] ethylenediamine (Q-EDDnp) was a kind gift of Prof. Dr. Maria A. Juliano (Escola Paulista de Medicina, Universidade Federal de São Paulo, Brazil). Phosphatidylinositol phosphates (PtdInsPs) and lipid strips were from Echelon Biosciences. Unfractionated heparin (ufh) with an average molecular weight of 18 kDa, sodium triphosphate pentabasic (polyP_3_) and sodium phosphate glass, type 45 (polyP_45_) were from Sigma-Aldrich. Enoxaparin (Clexane^TM^) was from Sanofi. Recombinant human vaspin wild type and variants as well as human KLK7 was expressed in and purified from *E. coli* as described previously [20]. The non-heparin binding (NHB) vaspin variant K188A/K131A/R211A/K359A/R363A was generated by site directed mutagenesis, sequentially adding the mutations for the amino acid exchanges R363A, K131A and K188A to the previously described R211A/K359A variant [25].

### 4.2. Lipid Overlay Assays

Hydrophobic membranes (spotted with 100 pmol of various lipid species per spot) or PtdIns arrays (with serial dilutions of different immobilized PtdIns ranging from 100 to 1.56 pmol/spot) were blocked with Pierce^TM^ Protein free (TBS) blocking buffer (Thermo Scientific, Waltham, MA, USA) at room temperature for 1 h. Then, membrane lipid strips were incubated with 1 µg/mL vaspin at room temperature for 1 h. Incubation with mouse anti-vaspin antibody VP63 (AdipoGen) was followed by anti-mouse antibody (CST#7076P2, Cell Signaling Technology, Danvers, MA, USA). Bound protein was detected via enhanced chemiluminescence using a Gbox documentation system (Syngene, Bangalore, India).

### 4.3. Complex Formation 

Complex formation of vaspin and KLK7 was performed as previously described [20]. Vaspin was incubated with 3 µM recombinant KLK7 (molar ratio protease/serpin 3:1) in the presence or absence of PtdInsPs (molar ratio PtdInsPs/vaspin 0.1, 1 or 10), ufh (molar ratio ufh/vaspin 10) or polyP (polyP_3_ or polyP_45_ 1−200 molar excess) for 1 min.

Proteins were separated using SDS-PAGE and gels were stained using Coomassie Brilliant Blue. Band intensities were determined by densitometric quantification (Gene tools software, Syngene) and were normalized to the reaction without PtdIns, ufh or polyP. Stoichiometry of inhibition (SI) was estimated from band intensities as previously described [20].

### 4.4. Kinetics

In order to determine inhibition rates of vaspin for KLK7, a discontinuous assay was applied as described before [20]. Briefly, commercial KLK7 was activated according to the manufacturer’s protocol. Inhibition of KLK7 by vaspin was measured under pseudo-first-order conditions using 19.2 nM KLK7, vaspin, PtdInsP/ufh/polyP (molar ratio 1:10:100) and 30 µM fluorogenic peptide NFF3 or 9 µM ABZ. Residual KLK7 activity was measured after 1 min (in the presence of ufh or polyP) or after 20 min of incubation time on a FlexStation3 Multi-Mode Microplate Reader (Molecular Devices, San Jose, CA, USA). The second-order rate constants were determined as described previously [20] and normalized to the reaction without ligand. 

### 4.5. Microscale Thermophoresis (MST)

MST measurements were performed as described previously [25]. Human vaspin and vaspin variant K188A/K131A/R211A/K359A/R363A were labeled using the Monolith NT protein amine-reactive labeling kit RED-NHS (NanoTemper Technologies, Munich, Germany). Average labeling efficiency was 1 and labeled proteins were stored in the MST buffer (50 mM Tris, 150 mM NaCl, 10 mM MgCl_2_ and 0.05% Tween 20, pH 7.6) at −20 °C. Labeled vaspin proteins were titrated with serial dilutions of enoxaparin (2.6 nM–1.4 mM in MST buffer with 0.1% (w/v) BSA) or polyP_45_ (4.7 nM–39 µM in MST buffer). Binding of enoxaparin and polyP_45_ was measured using 100 nM or 50 nM of labeled protein, respectively. All measurements were performed at room temperature in premium capillaries (NanoTemper Technologies) at least in triplicates. Data was analyzed using NanoTemper analysis software and GraphPad Prism7 (GraphPad) determining thermophoretic mobility. The fitting function is derived from the law of mass action as previously described in detail [67,68].

### 4.6. Nano Differential Scanning Fluorimetry (NanoDSF)

Thermal stability was determined using the Prometheus NT.48 device as previously described [69]. Protein was loaded into nanoDSF standard capillaries at a concentration of 0.1 mg/mL. Protein solutions were heated from 20 to 95 °C. Unfolding was measured monitoring intrinsic tryptophan and tyrosine fluorescence at 330 and 350 nm. 

### 4.7. Statistical Analysis

Data are presented as means ± SEM. Statistical analyses were performed using GraphPad Prism7 (GraphPad). Statistical significance was determined by Student’s two-tailed t-test or by one-way ANOVA followed by Dunnett’s post-hoc test when comparing multiple groups. *p* values ≤ 0.05 were considered to be significant.

## Abbreviations

KLK7	kallikrein 7
MST	microscale thermophoresis
PtdInsP	phosphatidylinositol phosphate
RCL	reactive center loop
PCI	protein C inhibitor
polyP	polyphosphate
ufh	unfractionated heparin
